# Immunity: Psoriasis comorbid with atherosclerosis

**DOI:** 10.3389/fimmu.2022.1070750

**Published:** 2022-12-15

**Authors:** Chunping Liu, Huiqi Chen, Yanjiao Liu, Haiding Huang, Wanling Yu, Tingting Du, Xinyao Long, Xinming Chen, Zhijun Chen, Sien Guo, Jinxin Li, Zebo Jiang, Lei Wang, Chuanjian Lu

**Affiliations:** ^1^ State Key Laboratory of Dampness Syndrome of Chinese Medicine, The Second Affilliated Hospital of Guangzhou University of Chinese Medicine, Guangzhou, China; ^2^ Guangdong-Hong Kong- Macau Joint Lab on Chinese Medicine and Immune Disease Research, The Second Affilliated Hospital of Guangzhou University of Chinese Medicine, Guangzhou, China; ^3^ Guangdong Academy of Chinese Medicine Sciences, Guangdong Provincial Hospital of Traditional Chinese Medicine, Guangzhou, China; ^4^ State Key Laboratory of Quality Research in Chinese Medicine, Institute of Chinese Medical Sciences, University of Macau, Macau, Macau SAR, China; ^5^ Guangdong Provincial Key Laboratory of Biomedical Imaging and Guangdong Provincial Engineering Research Center of Molecular Imaging, The Fifth Affiliated Hospital, Sun Yat-sen University, Zhuhai, Guangdong, China

**Keywords:** comorbidity, immunology, psoriasis, atherosclerosis, inflammation

## Abstract

Psoriasis is an immune-mediated, persistent inflammatory disease with a genetic predisposition, and the involvement of multiple organs in psoriasis remains indicative of systemic disease. Atherosclerosis (AS) is a common complication of patients with severe or prolonged psoriasis. The specific pathogenesis of psoriasis is still unclear. Current studies suggest that psoriasis is a polygenic genetic disease with the interaction of multiple factors such as heredity and environment. Keratinocytes are proliferated through immune-mediated inflammatory pathway, which leads to cell activation, infiltration of dermis cells and release of inflammatory factors. Activation of inflammatory cells and pro-inflammatory factors play an important role in the progression of psoriasis and atherosclerosis. Studies have found that there is a close relationship between psoriasis and atherosclerosis, and systemic inflammation may be the common feature of psoriasis and AS. This paper attempts to explore the possibility of the relationship between psoriasis and atherosclerotic comorbidities from the aspects of potential epidemiology and immune mechanism, in order to provide some reference for the subsequent scientific research.

## Introduction

Psoriasis is an immune-mediated persistent inflammatory disease with genetic tendency. The cutaneous lesions in psoriasis present as sharply demarcated erythema with white scales, and its pathological manifestations are chronic inflammation and abnormal proliferation and differentiation disorders of keratinocytes (1). Psoriasis is not contagious, but the pain and disability caused by psoriasis cause a heavy economic and psychological burden on patients, and seriously affect the quality of life (2). According to the announcement on the website of the American Psoriasis Foundation in 2020, there are more than 125 million psoriasis patients worldwide (3). Psoriasis occurs all over the world, but the incidence is uneven, with a high prevalence in people at high latitudes far from the equator, and a low prevalence in equatorial or equatorial Asian and African countries (4). The psoriasis described in this review refers to plaque psoriasis, which is the commonest subtype of psoriasis, accounting for 90% of all cases (5). The pathogenesis of psoriasis has not been fully elucidated, and the overactivation of adaptive immune system is considered to be the core of the pathogenesis of psoriasis. Dendritic cells, keratinocytes, macrophages and other immune cells secrete large amounts of interleukin, and induce the differentiation of primary T cells into T-helper cells Type 1 (Th1), and at the same time increase the number of Th17 and Th22 cells and prolong the survival time. Finally, tumor necrosis factor-α (TNF-α), IL-17, IL-22 and other pro-inflammatory factors are secreted, which activate the transcription of keratinocyte inflammatory factors (5). The skin is the main lesion of psoriasis, but the multiple organ involvement that leads to psoriasis is still indicative of systemic disease (1), and atherosclerosis (AS) is a common complication in patients with severe or prolonged psoriasis (5, 6).

AS is a vascular pathological change in which lipid and fibrous substances deposit in the intima of large and medium arteries, resulting in lumen narrowing or stimulating thrombosis, and ultimately leading to tissue ischemia. AS is the main pathological cause of Arteriosclerotic Cardiovascular Disease (ASCVD), which can lead to acute coronary syndrome, stroke, peripheral artery obstruction and other fatal or disabling diseases (7, 8). In addition to low density lipoprotein level, the pathological mechanism of AS is also related to plaque local inflammation level (9, 10). Elevated levels of coronary artery inflammation can lead to local inflammatory cell infiltration of atherosclerotic plaques, increasing their vulnerability and instability, and increasing the incidence of ASCVD adverse events (11, 12). Oxidized low density lipoprotein (oxLDL) is presented to antigen-presenting cells in atherosclerotic plaques. Toll-like receptor 4 (TLR4) and CD36 receptors on APC recognize and promote the release of inflammatory factors (13). CD4+T cells recognize apolipoprotein B presented by major histocompatibility complex II (MHC-II), induce Th1 and Th17 lymphocytes to differentiate, and secrete interferon -γ (IFN-γ) and IL-17. Thus increasing the inflammatory activity and instability of AS plaques. In addition, CD8+T cells can also recognize apolipoprotein B presented by MHC- ι on APC, resulting in the production of IFN- γ and the intensification of AS process (7). Thus, systemic inflammatory response is the common feature of psoriasis and AS.

## The epidemiology between psoriasis and coronary heart disease events

Psoriasis is closely related to the lesion of coronary artery. The study found (14) that patients with psoriasis had a higher incidence of cardiovascular events than patients without psoriasis and an increased risk of myocardial infarction, especially in patients with severe psoriasis and patients with early onset psoriasis. It is suggested that psoriasis increases the risk of coronary artery disease in patients. In this chapter we will detail the epidemiological studies between the two.

Guidelines for the prevention of cardiovascular disease (15) indicate an increased risk of cardiovascular disease in patients with psoriasis. A systematic review (14) of 33 observational controlled studies found that compared with the normal population, the incidence of cardiovascular events in patients with psoriasis was 1.25 and 1.57, respectively, and the risk of myocardial infarction was increased, especially in patients with severe psoriasis and early onset psoriasis. In another study (16), 323 patients with psoriasis had a 2.2 times higher risk of developing arterial and venous vascular disease (myocardial infarction, thrombophlebitis, pulmonary embolism, etc.) than 325 patients with other skin diseases. These epidemiological studies of cardiovascular disease and psoriasis reported that psoriasis was associated with the incidence of major cardiovascular disease (CV) events.

In addition, the incidence of cardiovascular events in psoriasis is also related to the severity of psoriasis. Studies have shown that psoriasis increases Framingham risk score by 6% (17), and the OR score for atherosclerosis is 2.18 (95%CI, 1.59-3.01), indicating an increased prevalence of atherosclerosis in patients with psoriasis compared with non-psoriatic patients (18). Arterial hardness is increased in patients with psoriasis, and hardness is positively correlated with the course of psoriasis (19), among which the impairment of coronary artery microvascular function is particularly severe in patients with severe psoriasis (20). CT results of another study also showed increased coronary artery calcification (CAC) (59.4% vs. 28.1%, P =0.015) and calcification severity (Agatston score 3.7 vs. 0.0, P =0.019) in patients with psoriasis, and increased with the severity of psoriasis (21).

Taken together, these studies show an association between psoriasis and cardiovascular disease, and recognition of an elevated risk of cardiovascular disease in patients with psoriasis is expected to spur further research.

## Immunity and psoriasis - atherosclerosis are closely related

**Figure 1:**
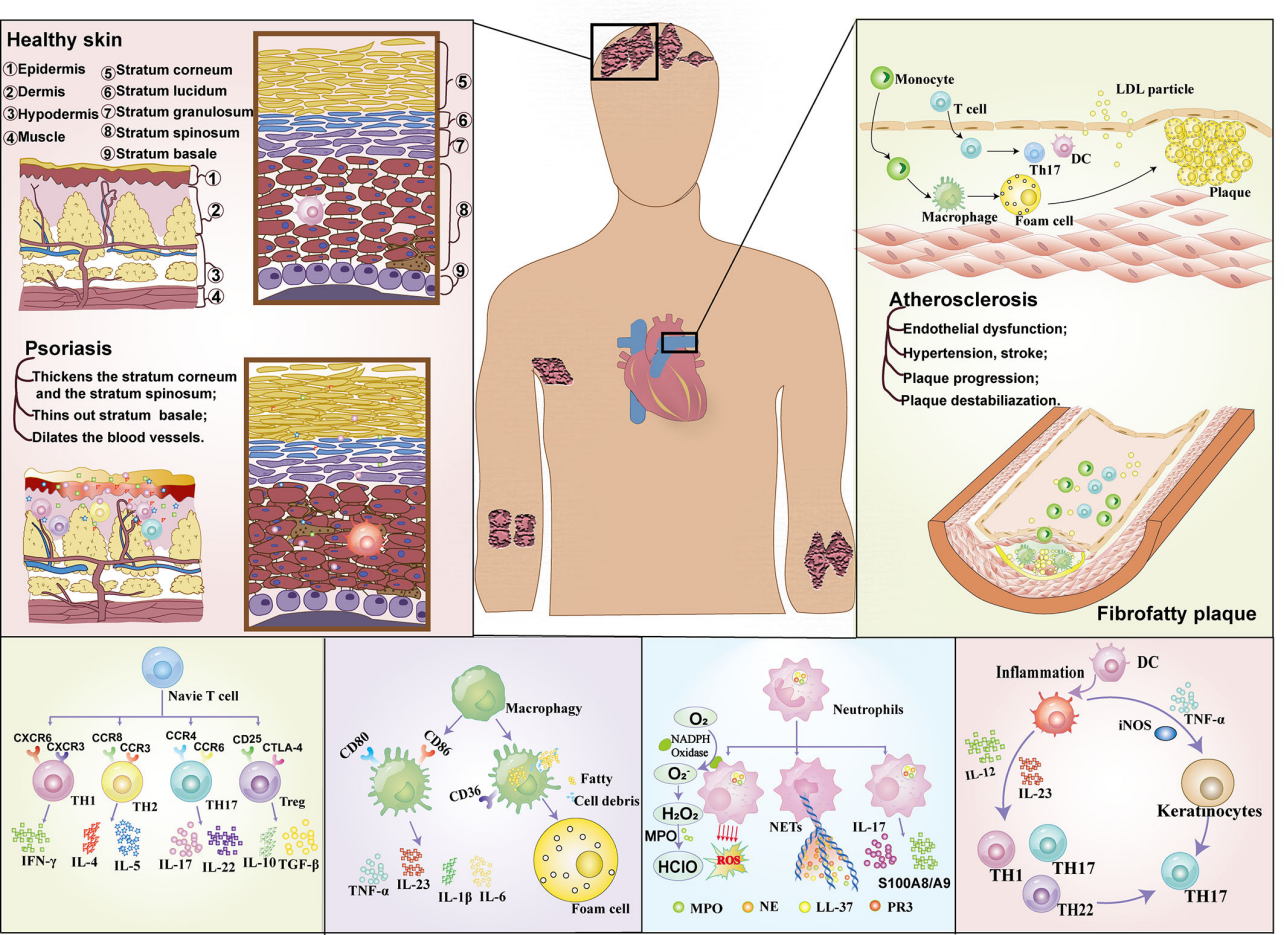
The immune mechanism of Psoriasis comorbidities with atherosclerosis.

In recent years, the immune association between psoriasis and atherosclerosis has attracted more and more attention (**Figure 1**). In the past, psoriasis was generally believed to be a disease mediated only by Th1 cells, but now it has been considered to be an inflammatory disease mediated jointly by Th1 and Th17 (22), and the similarity of immune pathogenesis spectrum (Th1/Th17) between the two (23) has gradually become a research hotspot. High levels of Th17-secreted cytokines in psoriasis patients (22) mediate vascular inflammation and the development of atherosclerosis (24, 25). In addition, helper T cells, regulatory T cells (Treg cells), dendritic cells, monocytes/macrophages and neutrophils (17) also play important roles in the progression of psoriasis and atherosclerosis (17, 26).

### T cells

T lymphocyte subsets are derived from lymphoid stem cells of bone marrow, which differentiate and mature into T cell subsets that recognize various antigens under the induction of thymus. They are distributed to immune tissues and organs of the whole body through lymphatic circulation and blood circulation, exerting cellular immunity and immune regulation functions. Early evidence suggests that both AS and psoriasis are th1-mediated diseases (26). The Th1 subtype is the most studied cell type in psoriasis at present, but the role of other T cell subtypes in different stages of psoriasis and atherosclerosis should not be ignored (27). Recent studies have found that Th17, CD8+T cells and γδT cells can produce IL-17 and participate in the pathogenesis of psoriasis and atherosclerosis (1).

Th1 is the key to the beginning of the inflammatory cascade in psoriasis (28) and activates keratinocytes through activation of neutrophils, macrophages and CD8+ cytotoxic T lymphocytes (29). The main mediators of Th1 activity are IFN-γ and IL-2, which act on keratinocytes and induce the production of antimicrobial peptides, leading to an inflammatory cascade. Elevated Th1 cells in patients with unstable angina and acute coronary syndrome (ACS) suggest that Th1 cells also play a key role in atherosclerosis, which is thought to be driven primarily by the cytokine IFN-γ that is the hallmark of the Th1 response. Therefore, the common feature between psoriasis and AS is that IL-12 stimulates the activation of Th1 response, which in turn activates the corresponding immune cells to produce an inflammatory cascade that affects subsequent endothelial dysfunction (17). In addition to the classic Th1 and Th2 cytokines, recently discovered members of the IL-17 cytokine family play important roles in regulating cellular and humoral immune responses.

Th17 cells in psoriasis process release different cytokines such as IL-17, IL-22 and tumor necrosis factor -α (30) and participate in the process of macrophage stimulation of dendritic cells (DC) to spread inflammatory response. Increased the release of inflammatory mediators such as monocyte chemotactic protein (McP-1), nitric oxide and vascular endothelial growth factor, suggesting that Th17 plays a positive role in promoting the inflammatory response in psoriasis. Compared with patients with stable ACS, Th17 cells and IL-17 levels were increased in patients with ACS (17, 31). Christian Erbel et al. established AS mice with high fat diet, and inhibition of IL-17A significantly reduced the atherosclerotic lesion area (P < 0.001), maximum stenosis (P < 0.001) and lesion vulnerability. Mice treated with IL-17A mab had reduced cell infiltration, down-regulated activation markers (e.g. Vcam-1) on endothelial cells and immune cells, and reduced cytokine/chemokine secretion (e.g. IL6, TNF-α, CCL5). It is suggested that IL-17A plays a role in AS through its extensive pro-inflammatory and pro-apoptotic effects (32, 33). However, there are different conclusions about the role of Th17 cells in AS (34). Onno J de Boer et al. (35) found that IL-17A/F(+) neutrophils were more common in complex plaques than in intact plaques. No IL-17A/F(+) T cells (TH17) were observed. Il-17e is expressed in normal and atherosclerotic smooth muscle cells and endothelial cells, and is also widely expressed in mature B cells in advanced plaques. Both cultured smooth muscle cells and endothelial cells expressed IL-17E and its functional receptor (IL-17Rb). Suggesting that the additional presence of IL-17E(+) B cells and IL-17A/F(+) neutrophils in advanced and complex plaques suggests that the complex contribution of IL-17 family cytokines to human atherosclerosis may depend on the stage and activity of the disease. In summary, further studies are needed to prove whether Th17 can promote atherosclerosis.

In addition, regulatory T cells (Treg) have also been found to be associated with psoriasis and AS. Treg is a subset of T lymphocytes, whose main function is to inhibit the activation and proliferation of T cells through mechanisms driven by cell-contact-dependent and cell-contact-independent anti-inflammatory cytokines (36). The inhibitory function of Treg was significantly impaired in patients with psoriasis, leading to chronic auto-inflammation in patients with psoriasis. The level of Treg in patients with cardiovascular disease is reduced (5), and the reduction in the number of Treg cells or their reaction ability can lead to the progression of atherosclerosis (37). It can be seen that the inflammatory process of psoriasis and atherosclerosis has a certain correlation with the decrease of Treg level.

### Macrophages

Macrophages are involved in host defense, dynamic homeostasis, and tissue repair. They can be divided into monocytes or tissue-derived macrophages according to the development degree (38), which play a key role in the pathogenesis of various immune-mediated diseases and chronic inflammation (39). Macrophages have traditionally been divided into pro-inflammatory M1 subtypes and anti-inflammatory M2 subtypes. Macrophages have strong heterogeneity and can be adjusted to adapt to the surrounding environment according to the pathological state (40–42). Macrophages are closely related to the development of psoriasis - atherosclerosis comorbidities.

Psoriasis is an immune-mediated inflammatory disease in which inflammatory cells promote the development of psoriatic lesions and atherosclerotic plaques. In psoriasis, macrophages acquire a pro-inflammatory phenotype and produce cytokines such as IL-23, TNF-α, and IL-1β. Il-23 and IL-1β activate T cells to produce cytokines such as IL-17 and IFN-γ that promote further polarization of macrophages, resulting in a sustained inflammatory state (38, 43). TNF-α can induce the expression of endothelial cell adhesion molecules that can promote the adhesion between monocytes and endothelial cells, and thus promote AS (44). In addition, injured endothelial cells can also secrete vascular endothelial growth factor (VEGF), which promotes the formation of vascular plaque by stimulating the aggregation of macrophages in arterial endothelial cells (18). Superoxide dismutase 2 (SOD2) is essential in the regulation of macrophage function and anti-oxidative stress (45), which helps to alleviate the damage of reactive oxygen species (ROS) to cells. Yvonne et al. found in psoriatic mice that the expression of SOD2 in macrophages was reduced by 60%, which may lead to enhanced oxidative stress in cells, aggravate cell damage and promote the formation of atherosclerotic plaques (45). In addition, macrophages in patients with psoriasis secrete increased Microparticles (MPs), which promote systemic inflammatory response and coagulation disorders, which may affect the thickness of intima and middle layer and the curvature of capillaries in nail folds, thus promoting the progression of atherosclerosis (46, 47). In conclusion, macrophages promote and maintain inflammatory response and promote the development of AS in patients with psoriasis. In addition, macrophages not only promote the expression of inflammatory factors in patients with psoriasis, but also increase the formation of cholesterol crystals and lipid uptake, affect cholesterol excretion, and further promote the occurrence of AS (45, 48, 49).

In conclusion, macrophages are involved in the disease process of psoriasis and AS and play a crucial role, but further studies are still needed to clarify their mechanisms.

### Neutrophils

The role of neutrophils in atherosclerosis and psoriasis has also been gradually recognized. Animal models and clinical trials have proved that their common mechanism of action is that neutrophils drive excessive proliferation of keratinocytes through IL-17, leading to chronic skin inflammation (17). Neutrophils act as important defenders in acute inflammatory responses, eliminating foreign pathogens by releasing reactive oxygen species and proteolytic enzymes that may also lead to tissue destruction. In addition, neutrophils have a variety of biological functions in innate immunity, and neutrophils can synthesize and release cytokines, chemokines and growth factors after stimulation (50). When these factors interact with damaged endothelial cells, neutrophils release granular proteins that increase leukocyte recruitment in the endothelial layer and ultimately promote inflammation and foam cell development, leading to further development of atherosclerosis and psoriatic inflammation (51).

Psoriasis increases neutrophil activation and the release of neutrophil associated proteins, such as calcium-binding proteins (S100A8/A9), which may further provide a link between psoriasis and cardiometabolic disease. S100A8/A9 is released by activated neutrophils and upregulated in psoriatic lesions (50). Studies (50) have proved that S100A8/A9 is closely related to the severity of skin diseases and vascular inflammation (VI). In conclusion, neutrophils and their proteins may be involved in the early atherosclerotic environment of psoriasis and independently predict endothelial dysfunction. With the development of various studies, a new subtype of neutrophil – low density granulocyte (LDG) is becoming a hot research direction in the pathophysiology of psoriasis and cardiovascular diseases. LDG is characterized by high pro-inflammatory activity, changes in phagocytosis, mediated production of type I interferon, and high abundance in atherosclerotic plaques and plasma in patients with psoriasis (50). LDG differs from autologous normal density granulocytes (NDG) and healthy control neutrophils (52, 53) in gene expression level, and LDG is also different from NDG in phenotype. Among these differences, the most striking was their increased ability to spontaneously form neutrophil extracellular traps (NETs). This new subtype of neutrophil will provide more direct evidence of the link between psoriasis and cardiometabolic disease.

### Other immune cells

The inflammatory microenvironment of psoriatic lesions and early atherosclerotic plaques, including chemokines and cytokines secreted by dendritic cells and NK cells in immune cells like immune cells, can promote the inflammatory environment and lead to the formation of psoriatic plaques or atherosclerotic plaques.

Dendritic cells (DC) not only serve as antigen presenting agents and cellular gene producers, but also as Bridges between innate and adaptive immune systems, and play an important role in continuing chronic inflammation-induced cascades (54). For example, plasmacytoid dendritic cells (pDCs) are involved in type 1 interferon (IFN) response (28, 55), while dendritic cells (mDCs) from bone marrow are key cells in the production of IL-12 and IL-23, thus inducing the expansion of specific Th cells. These two DCS play an important role in the formation of psoriatic inflammation. Although new evidence suggests that DC also plays a role in atherosclerotic plaque formation and regulates atherosclerotic plaque vulnerability through cholesterol metabolism and adaptive immune responses (56), the joint role of DC in psoriasis and atherosclerosis needs further study.

NK cells are generally considered to belong to the lymphocytes of the innate immune system and can directly kill viruses/cancer cells, while also producing cytokines including IFN-γ and TGF-β. Ottaviani et al. (57) found that 5-8% of inflammatory infiltrating cells entering psoriatic skin were composed of CD56+ and CD3− cells in the NK cell phenotype, most of which were CD56bright subsets of NK cells. Compared to CD56dim NK cells, CD56bright cells are considered to represent more immature cells with less cytotoxicity, while simultaneously activating the antigen CD69 and producing large amounts of IFN-γ *in vitro*. IFN-γ is an important mediator of inflammation in psoriasis and atherosclerosis, stimulating the expression of MHC-II molecules and ICAM-1. Elevated serum IFN-γ in patients with psoriasis may stimulate the proliferation of keratinocytes, resulting in keratinization defects and changes in the biological characteristics of keratinocytes, resulting in typical psoriatic lesions (58). In addition, IFN-γ enhances antigen presentation, activates T lymphocytes, interacts with a variety of inflammatory factors, promotes inflammatory response at the atheroma site, and aggravates the progression of the disease. Therefore, cytokines produced by NK cells play an important role in inflammatory changes in psoriasis and atherosclerosis.

In conclusion, T cells, macrophages and neutrophils participate in the pathogenesis of psoriasis and AS, and play an important role in different stages of psoriasis and AS.

## Conclusion

In recent years, a large number of studies have explained the relationship between psoriasis and coronary atherosclerosis from different perspectives, but there are still many questions to be solved. The correlation between psoriasis and AS may be the result of multi-factor interaction. From the perspective of immunological pathogenesis, psoriasis and AS have similar immune-mediated inflammatory responses, and immune imbalance may be the common mechanism of both. Systemic inflammation of psoriasis may contribute to the increased cardiovascular risk of AS in many inflammatory diseases, but the exact mechanisms leading to AS may vary in different inflammatory diseases. To sum up, most literature reports and research results indicate that patients with psoriasis have an increased risk of central vascular disease, and immune imbalance may be the common pathogenesis of the two, which needs further study.

## Author contributions

CLi wrote the main piece. HC and YL drew the artwork. HH, WY and TD proofread the manuscript. XL, XC, ZC, SG and JL were responsible for the insertion of the literature, and ZJ, LW and CLu were responsible for the writing instruction and the embellishing. All authors contributed to the article and approved the submitted version.
